# Association between mobile phone use and risk of rheumatoid arthritis: A large prospective cohort study

**DOI:** 10.1371/journal.pone.0347330

**Published:** 2026-05-22

**Authors:** Shaoguang Ren, Peng Gao, Sitong Wei, Yang Cui, Dongqing Ye, Xinyu Fang

**Affiliations:** 1 Department of Epidemiology and Biostatistics, School of Public Health, Anhui Medical University, Hefei, Anhui, China; 2 Inflammation and Immune Mediated Diseases Laboratory of Anhui Province, Hefei, Anhui, China; 3 School of Public Health, Anhui University of Science and Technology, Hefei, Anhui, China; University of Texas UTHSCSA: The University of Texas Health Science Center at San Antonio, UNITED STATES OF AMERICA

## Abstract

**Background:**

It remains uncertain whether there is a relationship between mobile phone use and rheumatoid arthritis (RA). Research on the relationship between different mobile phone usage(MPU) behaviors and the risk of RA onset is still insufficient.

**Methods:**

The UK Biobank (UKB) data were utilised to inquire into the relationship between four mobile phone use exposure variables—MPU, length of mobile phone use(LMPU), weekly usage of mobile phone for making or receiving calls(WMPU), and hands-free device/speakerphone use with mobile phones to make or receive calls(HMPU)—and new-onset RA. The relationships between MPU behaviors and the occurrence of RA in the general population were assessed using Cox regression analyses. These associations were further explored in subgroups stratified. We implemented sensitivity analyses to confirm the stability of the results.

**Results:**

During a median follow-up period of 13.63 years, 6082 new cases of RA were identified among 479,966 participants. Individuals who used cell phones had a 14% elevated risk of the onset of RA (HR: 1.14, 95% CI: 1.07–1.23), with an 8% elevated risk (HR: 1.08, 95% CI: 1.02–1.15) observed among those who used a mobile phone for more than 30 minutes per week.

**Conclusions:**

We aimed to investigate the association between MPU and the risk of developing RA in the general population. Results indicate that increased MPU, longer duration of use, and greater weekly mobile phone usage time may be associated with an elevated risk of developing RA.

## Introduction

RA is an autoimmune disease characterized by bone erosion and progressive cartilage destruction [1]. Tumor necrosis factor-α (TNF-α) and interleukin-1β (IL-1β) are core inflammatory mediators that drive the pathological progression of RA. TNF-α and IL-1β activate leukocytes, endothelial cells, and synovial fibroblasts. Synovial fibroblasts produce large amounts of matrix metalloproteinases (MMPs), which degrade collagen and proteoglycans, key components of cartilage. Furthermore, TNF-α and IL-1β can also activate osteoclasts, promoting cartilage and bone resorption. The degradation of cartilage and bone resorption ultimately triggers RA [2]. By 2050, RA is projected to affect 31.7 million individuals, imposing a substantial burden on the global healthcare system [3]. Therefore, to enhance the primary prevention of RA, it is necessary to identify more modifiable risk factors.

Mathieu S et al. reported that smartphone use may be associated with aggravated hand pain symptoms in RA patients [4]. Meanwhile, due to the lack of research on the impact of mobile phone use on RA onset, and since multiple sclerosis (MS) shares the autoimmune disease classification with RA, a comparison between the two conditions was drawn. Khaki-Khatibi F et al. found that increased mobile phone use may be an independent risk factor for the progression of multiple sclerosis [5]. This has drawn our attention, and we aim to investigate whether mobile phone use could be a potential influencing factor for RA onset. Concurrently, by 2020, the global number of mobile phone users potentially affected was projected to reach 8.2 billion [6]. This has also raised significant concerns about the safety of MPU, with heavy users being a group of particular concern. Studies by Lu Y et al. indicate that prolonged exposure to radiofrequency electromagnetic fields (RF-EMF) and blue light emitted by mobile phones leads to elevated levels of inflammatory mediators such as TNF-α and IL-1β. When human brain microglia and astrocytes were exposed to 1800 MHz RF-EMF, the concentration of inflammatory cytokines increased significantly [7]. Wistar rats exposed to 1966.1 MHz RF-EMF also exhibited elevated levels of the inflammatory cytokine TNF-α [8].

Furthermore, research by Berson D M et al. and Nah SS et al. revealed that 484 nm blue light emitted by mobile phones can activate retinal ganglion cells, thereby suppressing pineal melatonin secretion. Melatonin itself inhibits the expression of TNF-α and IL-1β [9, 10]. This suggests that nighttime mobile phone use may elevate the concentrations of TNF-α and IL-1β within the body. Inflammatory mediators such as TNF-α and IL-17 activate synovial fibroblasts, osteoclasts, and chondrocytes, leading to bone resorption, bone erosion, and cartilage destruction—key pathways in the pathogenesis of rheumatoid arthritis [2]. Given that both the radiofrequency electromagnetic fields and blue light emitted by mobile phones are implicated in activating these inflammatory mediators, it is reasonable to hypothesize a potential association between mobile phone use and an increased incidence of RA.

Based on the above hypothesis, we aim to investigate the impact of mobile phone use and its usage patterns on the development of RA. The innovation of our study lies in being the first to systematically utilize the UK Biobank database to examine the association between mobile phone use and the risk of developing rheumatoid arthritis in the general population. By analyzing the influence of different mobile phone usage characteristics on RA risk, this research provides a pathway for understanding the link between mobile phone use and autoimmune diseases.

## Materials and methods

### Research population

The UKB is a follow-up study. From 2006 to 2010, UKB recruited over 500,000 attendees at 22 assessment centers. At enrolment, these attendees completed nurse-led interviews and touchscreen questionnaires. This article describes the queue design and data collection methods in detail [11].

**Fig 1:**
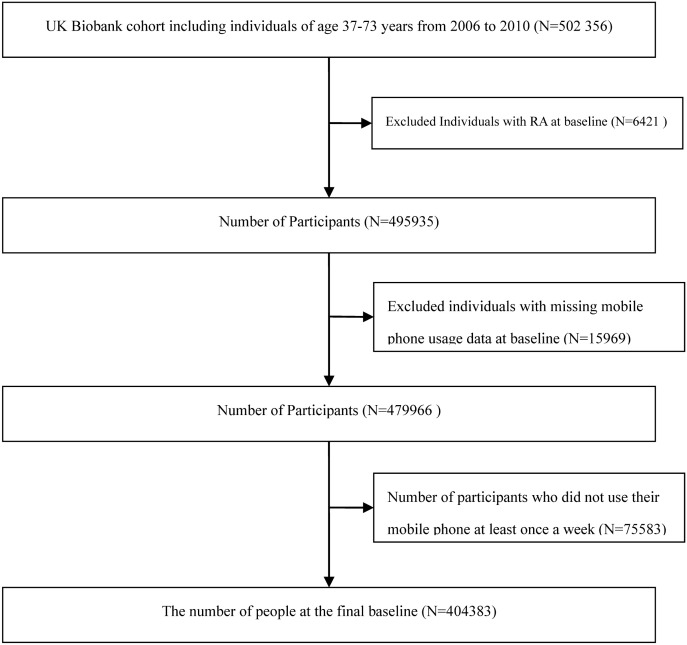
Participant Screening Flowchart..

The diagnosis of disease onset was determined by accessing the UKB database using RA disease codes during the follow-up period. Participants with RA at baseline were first excluded from the current study, followed by the deletion of those with missing information on MPU behaviors **(Fig 1)**.

We screened the participants entering the cohort by first excluding those with prevalent rheumatoid arthritis at baseline, followed by the exclusion of participants with incomplete mobile phone use information. Finally, individuals with incomplete data on weekly mobile phone usage time and hands-free device use were excluded. This resulted in a final cohort of 479,966 participants with complete mobile phone use information, and 404,383 participants with complete data on both weekly mobile phone usage time and hands-free device use.

Prior to entering the queue, informed consent was obtained from all participants.. And the Northwest Research Ethics Board approved the ethical request for UKB(Ref: 11/NW/0382).

### Measurement of exposure variables

Four mobile phone usage characteristics—(MPU, LMPU, WMPU, and HMPU)—were derived from responses to three touchscreen questionnaire items completed by UKB participants at baseline (2006–2010) [12]. The classification of the four MPU behaviours examined in our study can be found in **S1 File**, **and** **S1 Table**.

### Ascertainment of outcomes

The study outcome was RA. Cases of RA were identified based on codes from International Classification of Diseases version(ICD)-9, ICD-10, and self-reported data. Detailed codes are provided in **S2 Table****.** Determination of follow-up time was calculated using the following methodology: the starting point of the calculation was the date of entry into the cohort, and the end point of the calculation was the date of occurrence of RA or follow-up loss date, death, or the expiration date (October 31, 2022) [13].

### Covariate measurements

The following variables were considered potential covariates: age (continuous variable), standard polygenic risk score for RA(continuous variable, RAPRS), drinking frequency (daily or almost daily, 3–4 times a week, 1–2 times a week, 1–3 times a month, only on special occasions or never), gender (male or female), sleep quality (low, medium, excellent) [14], qualification (university degree or other), race(white or other), smoking status(never, previous, current), Townsend deprivation index(continuous variable, TDI) [15]and body mass index (BMI, continuous variable), with BMI calculated as BMI = weight (kg) / height² (m²) [16].

RAPRS data from UKB's RAPRS was downloaded directly. And PRS was calculated by a Bayesian algorithm incorporating both multiple ancestry and related trait data. The calculation of the individual polygenic risk score (PRS) involved summing, across the genome, the product of the posterior effect size of each variant and its allele dosage [17,18].

For detailed information, please refer to the UKB online protocol (www.ukbiobank.ac.uk) for specific covariate measurements and **S2 File**.

### RA treatment medications and their codes

Records of RA treatment medications used by patients were collected through oral interviews conducted by well-trained nurses. During these interviews, detailed RA-specific information was recorded as free text and later converted into unique coded data. RA-related treatment medications include steroids, synthetic disease-modifying antirheumatic drugs (DMARDs), and biologic DMARDs, among others. Specific codes are provided in **S3 Table** [13].

### Statistical analysis

The LMPU was used as a basis for crowd segmentation. Baseline characteristics were compared using analysis of variance for continuous variables (presented as mean ± standard deviation) and chi-square tests for categorical variables (presented as proportions). The number and percentage of participants with missing covariates are shown in **S4 Table**. Missing values were imputed using the R package “mice” with 5 imputations. The variables included in the imputation were Education, RAPRS, BMI, Smoking, Race, TDI, Drinking, Sleep quality, Age, and Sex (10 variables in total).

We employed Cox regression analysis to evaluate the impact of MPU (compared to non-users) and LMPU on RA in the general population. Furthermore, among mobile phone users only, the associations between RA incidence and two usage metrics were assessed: WMPU and HMPU. The covariates adjusted for Model 1 included BMI, age, sex, smoking status, TDI, drinking frequency, and qualification. Sleep quality and race were adjusted in Model 2, on top of Model 1. Model 3 further incorporated interaction terms for different call usage behaviors based on Model 2. The specific inclusion procedure was as follows: during Cox regression analysis involving participants with complete data on both WMPU and HMPU variables, when performing Cox regression for the WMPU variable, HMPU was added to Model 2; conversely, when performing Cox regression for the HMPU variable, WMPU was added to Model 2. RAPRS was directly included in the model in both cases. We used the Schoenfeld residual method to test whether the data satisfied the proportional hazards assumption of the Cox model.

We conducted a dose-response analysis between WMPU and RA. WMPU was classified into six ordered categories (<5 minutes, 5–29 minutes, 30–59 minutes, 1–3 hours, 4–6 hours, > 6 hours). To analyze it as a continuous variable, we applied the midpoint method to convert it into a continuous scale (corresponding to 2.5, 17, 45, 120, 300, and 420 minutes, respectively). Three complementary analytical approaches were employed: (1) Linear trend analysis: simple linear regression was used to test for a monotonic dose-response relationship; (2) Nonparametric smoothing: locally weighted scatterplot smoothing (LOESS) was applied with a smoothing parameter (span) set to 0.75 and local quadratic polynomial fitting, allowing identification of potential nonlinear patterns without assuming a predefined functional form; (3) Categorical estimates: hazard ratios for each exposure category were presented as a reference benchmark. Statistical significance of the linear trend was assessed using the Wald test, while deviation from linearity was evaluated by comparing linear and quadratic models using likelihood ratio tests.

Additionally, subgroup analyses were implemented by age (<60 years, ≥ 60 years), drinking frequency, BMI (<30, ≥ 30), gender (male or female), sleep quality (low, medium, excellent), and qualification (university degree or other), to test whether significant interaction effects existed between these subgroup variables and two mobile phone usage metrics: MPU and WMPU.The classification of drinking frequency is detailed in the covariates measurements section [11].

To ensure the reliability of the results, we performed sensitivity analyses as follows: Sensitivity Analysis 1 excluded cases of rheumatoid arthritis (RA) diagnosed within 5 years after enrollment, Sensitivity Analysis 2 further adjusted for physical activity and pre‑tax income variables [19], Sensitivity Analysis 3 excluded all participants with any missing data; Sensitivity Analysis 4 defined RA cases as those with either an ICD‑9 or ICD‑10 diagnosis code plus self‑reported RA and concurrent use of RA medications, and analyzed them as total RA cases [20], Sensitivity Analysis 5 included only seropositive RA cases and excluded other incident cases from the original study, Sensitivity Analysis 6 treated the grouping variables MPU and WMPU as numeric variables for analysis (reference) [6]. All analyses were performed using R version 4.4.1 with two-sided tests, and the significance threshold was set at p = 0.05.

## Results

### Baseline characteristics of attendees

The final study population comprised 479966 participants (mean [SD] age, 56.48 [8.09] years, with the detailed screening process shown in **Fig 1**(220565 [45.95%] males and 259401 [54.05%] females) enrolled in this investigation. By racial composition, the cohort included 436941 White (91.04%) and 43025 other (8.96%) individuals. During a median follow-up of 13.63 years, 6082 new cases of RA were diagnosed. The baseline characteristics stratified by the LMPU are summarized in Table 1. Compared with non-users, mobile phone users were older, had a higher proportion of females, and possessed lower levels of education (P < 0.001).

**Table 1:** Baseline characteristics of mobile phone use duration among the general population.

Baseline features		Length of mobile phone use				P Value
	Never	≤1 year	2-4 years	5-8 years	>8 years	
N	72684	12971	83863	147342	163106	
Age, years	60.38 ± 6.99	58.23 ± 7.79	57.08 ± 7.82	55.97 ± 7.98	54.75 ± 8.16	<0.001
Sex, n (%)						<0.001
Female	38236(52.61)	7815(60.25)	54158(64.58)	87588(59.45)	71604(43.90)	
Male	34448(47.39)	5156(39.75)	29705(35.42)	59754(40.55)	91502(56.10)	
BMI, kg/m2, ± s	26.96 ± 4.78	27.28 ± 4.94	27.21 ± 4.8	27.27 ± 4.72	27.83 ± 4.77	<0.001
TDI, ± s	−1.46 ± 3.02	−0.88 ± 3.27	−1.2 ± 3.1	−1.34 ± 3.05	−1.42 ± 3.05	<0.001
PRS, ± s	0.13 ± 0.98	0.12 ± 0.97	0.13 ± 0.98	0.13 ± 0.99	0.12 ± 0.99	<0.001
Education, n (%)						<0.001
Others	42476(58.44)	8473(65.32)	55367(66.02)	92680(62.90)	98398(60.33)	
University degree	30208(41.56)	4498(34.68)	28496(33.98)	54662(37.10)	64708(39.67)	
Smoking, n (%)						<0.001
never	43516(59.87)	7508(57.88)	47723(56.91)	80277(54.48)	83977(51.49)	
previous	22403(30.82)	4045(31.18)	27965(33.35)	51980(35.28)	60220(36.92)	
current	6765(9.31)	1418(10.93)	8175(9.75)	15085(10.24)	18909(11.59)	
Drinking, n (%)						<0.001
Daily or almost daily	14762(20.31)	2031(15.66)	14060(16.77)	29031(19.70)	39125(23.99)	
3-4 times per week	14746(20.29)	2536(19.55)	18133(21.62)	35699(24.23)	41051(25.17)	
1-2 times per week	16811(23.13)	3318(25.58)	23351(27.84)	39793(27.01)	40785(25.01)	
1-3 times per month	8083(11.12)	1535(11.83)	10002(11.93)	16911(11.48)	17058(10.46)	
Special occasions only	10252(14.10)	1947(15.01)	10923(13.02)	15963(10.83)	15056(9.23)	
never	8030(11.05)	1604(12.37)	7394(8.82)	9945(6.75)	10031(6.15)	
Sleep quality, n (%)						<0.001
Low	2544(3.50)	522(4.02)	2855(3.40)	4762(3.23)	5553(3.40)	
Medium	31250(42.99)	5683(43.81)	35861(42.76)	63101(42.83)	72700(44.57)	
Excellent	38890(53.51)	6766(52.16)	45147(53.83)	79479(53.94)	84853(52.02)	
Race, n (%)						<0.001
Others	5257(7.23)	1276(9.84)	7128(8.50)	12680(8.61)	16684(10.23)	
White	67427(92.77)	11695(90.16)	76735(91.50)	134662(91.39)	146422(89.77)	

### Relationship between MPU, LMPU, and the prevalence of RA in the UKB attendees

MPU was positively correlated with an increased probability of developing RA (HR = 1.14, 95% CI: 1.07–1.23). Compared with non-users, those with ≤1 year of mobile phone use (HR = 1.19, 95% CI: 1.03–1.39), 2−4 years (HR = 1.11, 95% CI: 1.02–1.21), 5−8 years (HR = 1.14, 95% CI: 1.06–1.24), and >8 years (HR = 1.16, 95% CI: 1.07–1.26)(Table 2 **and** S1 Fig).

**Table 2:** The association between mobile phone use and weekly mobile phone use time with the RA occurrence hazard in the general population.

Mobile phone use			Model 1a	Model 2b	Model 3c
	N	Cases	HR (95% CI)	HR (95% CI)	HR (95% CI)
Length of mobile phone use (years)					
never	72684	951	ref	ref	ref
≤1	12971	208	1.20 (1.03-1.39)	1.19 (1.03-1.39)	–
2-4	83863	1172	1.11 (1.02-1.21)	1.11 (1.02-1.21)	–
5-8	147342	1896	1.14 (1.05-1.23)	1.14 (1.06-1.24)	–
>8	163106	1855	1.16 (1.07-1.26)	1.16 (1.07-1.26)	–
P_trend_			>0.05	>0.05	
Mobile phone use					
No	72684	951	ref	ref	ref
Yes	407282	5131	1.14 (1.07-1.23)	1.14 (1.07-1.23)	–
Weekly usage time of mobile phones for making or receiving calls					
<5 min	84115	1152	ref	ref	ref
5-29 min	157901	1982	0.98 (0.91-1.05)	0.98 (0.91-1.06)	0.98 (0.91-1.06)
30-59 min	69892	820	0.99 (0.90-1.08)	0.99 (0.90-1.08)	0.99 (0.90-1.08)
1-3 h	57969	703	1.12 (1.02-1.24)	1.12 (1.02-1.23)	1.12 (1.01-1.23)
4-6 h	16960	215	1.24 (1.07-1.44)	1.23 (1.06-1.43)	1.23 (1.05-1.42)
>6 h	17546	205	1.21 (1.04-1.40)	1.19 (1.02-1.38)	1.19 (1.02-1.39)
P_trend_			>0.05	>0.05	>0.05
Categories					
<30 min	242016	3134	ref	ref	ref
≥30 min	162367	1943	1.09 (1.03-1.16)	1.09 (1.02-1.15)	1.08 (1.02-1.15)
Hands-free device/speakerphone used for making or receiving calls					
Never or almost never	332062	4336	ref	ref	ref
Less than half the time	38273	394	1.08 (0.97-1.20)	1.08 (0.97-1.20)	1.05 (0.94-1.17)
About half the time	14832	147	1.06 (0.90-1.25)	1.06 (0.90-1.26)	1.02 (0.86-1.20)
More than half the time	8340	70	0.91 (0.72-1.16)	0.91 (0.72-1.16)	0.86 (0.68-1.10)
Always or almost always	10876	130	1.12 (0.94-1.33)	1.12 (0.94-1.33)	1.06 (0.89-1.27)
P_trend_			>0.05	>0.05	>0.05

aModel1: adjusted for age, BMI, sex, TDI, smoking status, alcohol frequency, qualification.

bModel2: adjusted for age, BMI, sex, TDI, smoking status, alcohol frequency, qualification, sleep quality, race.

cModel3: adjusted for covariates in Model 2 plus mutual adjustments for different MPU behaviors and RAPRS.

### Relationship between WMPU, HMPU, and the prevalence of RA among UKB mobile phone users

Increased WMPU was positively correlated with an increased probability of developing RA. Compared with participants using cell phones for < 5 minutes weekly, those using them for 1−3 hours (HR = 1.12, 95% CI: 1.01–1.23), 4−6 hours (HR = 1.23, 95% CI: 1.05–1.42), and >6 hours (HR = 1.19, 95% CI: 1.02–1.39) **(Table 2** **and** **S2 Fig****)**. Compared with those using devices for <30 minutes weekly, participants using devices for ≥30 minutes weekly showed an increased probability of RA occurrence (HR = 1.08, 95% CI = 1.02–1.15) **(Table 2)**.

Overall, among mobile phone users, the relationship between HMPU and the onset of RA was not statistically significant **(Table 2** **and** **S3 Fig****)**.

### Dose-response relationship between WMPU and RA incidence

There exists a clear nonlinear dose-response relationship between WMPU and the risk of RA onset (**S4 Fig**). The nonparametric LOESS smoothing curve (blue solid line) reveals a typical J-shaped pattern: at low to moderate usage levels (<1 hour/week), the hazard ratio remains close to the baseline (HR ≈ 1.0). When usage exceeds 1 hour/week, the risk begins to rise significantly, peaking at 4–6 hours/week (HR = 1.23). The > 6 hours/week group shows a slight decline in risk (HR = 1.19). In contrast, the linear trend line (red dashed line) indicates a consistent but gradual upward trend, failing to accurately capture the threshold effect. Categorical estimates (black dashed line) largely align with the LOESS curve, further confirming the presence of a nonlinear pattern. The linear trend test was not statistically significant (P > 0.05), whereas a formal test for nonlinearity (comparing linear vs. quadratic models) showed a significant deviation from linearity (P < 0.05).

### Subgroup analysis

**Fig 2:**
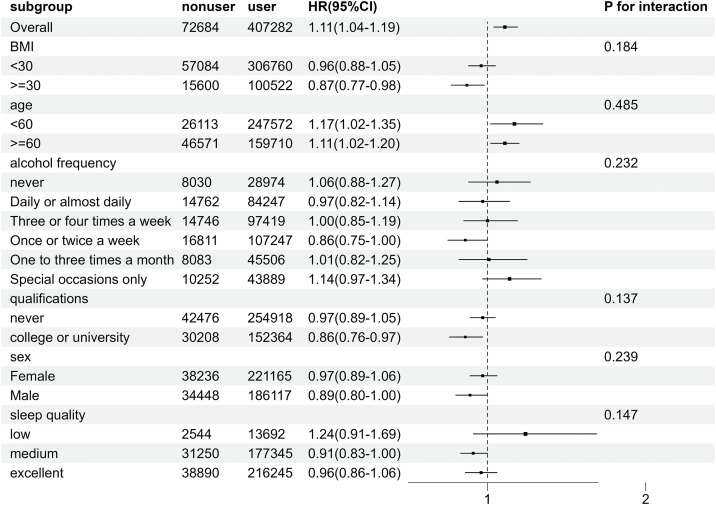
Subgroup analysis of factors influencing the link between mobile phone use and the incidence of new-occurrence Rheumatoid Arthritis.

**Fig 3:**
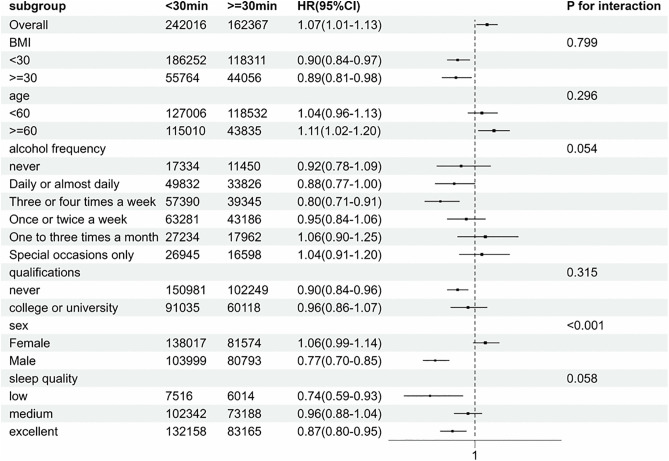
Subgroup analysis of factors influencing the link between weekly mobile phone use time and the incidence of new-occurrence Rheumatoid Arthritis.

Stratified analyses were conducted to test whether the relationships between new-onset RA and two exposure variables—(1) MPU (nonusers vs. users) and (2) WMPU (<30 minutes vs. ≥ 30 minutes)—were modified by various subgroup variables (Fig 2 **and** Fig 3).

In the subgroup analysis of the association between the WMPU and the onset of RA (Fig 3). Results stratified by gender indicated that WMPU ≥30 minutes was associated with RA risk, with a lower risk in males (HR = 1.04, 95% CI: 0.94–1.16) compared to females (HR = 1.16, 95% CI: 1.08–1.25) (**S5 Table**).

The relationship between MPU and the onset of RA was not modified by any of the studied variables—including BMI, qualifications, age, sleep quality, sex, or alcohol frequency.

We conducted subgroup analyses stratified by six variables—age, frequency of alcohol consumption, BMI, gender, sleep quality, and educational attainment—to assess whether there were significant interactions between these subgroup variables and mobile phone use. As illustrated in **Fig 2**, no significant interactions were detected between the subgroup variables and mobile phone use.

We performed subgroup analyses stratified by six variables—age, frequency of alcohol consumption, BMI, gender, sleep quality, and educational attainment—to examine whether significant interactions existed between these subgroup variables and weekly mobile phone use time. As shown in **Fig 3**, a significant interaction was observed between gender and weekly mobile phone use time, while no significant interactions were detected for the remaining variables.

### Sensitivity analysis

The results of the three sensitivity analyses remained relatively robust. In Sensitivity Analysis 1, after excluding participants with follow-up time ≤5 years, the results showed no significant changes **(****S6 Table****)**. Sensitivity Analysis 2, which conducted a complete-case analysis by removing all records with missing values, yielded consistent findings **(****S7 Table****)**. In Sensitivity Analysis 3, further adjustment for potential confounders (including pre-tax income and physical activity level) also did not significantly alter the results **(****S8 Table****)**. For Sensitivity Analysis 4, when defining RA cases as individuals meeting ICD-9 or ICD-10 diagnostic criteria, or self-reporting RA while concurrently using disease-modifying medication, the analysis of total RA incidence similarly showed no substantial changes **(****S9 Table****)**. Sensitivity Analysis 5, which included only seropositive RA cases and excluded the remaining incident cases from the original study, yielded results that were no longer statistically significant. This may be attributed to the reduced number of cases, leading to diminished statistical power **(****S10 Table****)**. Finally, Sensitivity Analysis 6, which treated the grouping variables MPU and WMPU as continuous variables for analysis, demonstrated no significant changes in the outcomes **(****S11 Table****)**.

## Discussion

In UKB, we assessed the association between four exposure variables—MPU, LMPU, WMPU, and HMPU—and the incidence of RA. We found a positive correlation between MPU, LMPU, and the onset of RA. The results revealed that in the general population, MPU and LMPU were positively associated with the incidence of RA. Among mobile phone users, weekly usage for making or receiving calls increased the probability of developing RA in the general population. However, there was no statistically significant relationship between the use of HMPU and the risk of RA onset. Furthermore, it was indicated by our findings that women constitute a high-risk group for increased RA incidence associated with mobile phone usage.

To our knowledge, the relationship between MPU and the incidence of RA remains unexplored. This research aims to address this gap. Given that both RA and MS are autoimmune diseases, we compared our findings with prior literature examining MPU and MS incidence. Given that the association between MPU and the incidence of RA has not been explored previously, we can only compare our findings with those of other autoimmune diseases. Since RA and MS are both autoimmune diseases, we compared our results with prior literature investigating the association between MPU and the incidence of MS. The association between MPU and MS was investigated by Aslak Harbo Poulsen et al in earlier work, with no statistically significant correlation being found between the number of mobile phone users and MS incidence or mortality in the general population [21]. Nevertheless, the present study indicates a positive correlation between MPU and increased RA incidence in the general population. One possible explanation lies in the differing methodologies employed to measure mobile phone usage across studies. In Harbo Poulsen et al.’s research, usage was measured by accessing records of all Danish mobile phone subscribers from 1982 to the end of 1995. This approach carries potential limitations: mobile service subscribers may not necessarily be actual users, and others may use the device on their behalf, leading to an underestimation of actual usage rates. In contrast, this study measured mobile phone usage by collecting self-reported data via touchscreen questionnaires completed by cohort participants. This method is more accurate and yields more reliable results.

Although the potential mechanisms linking MPU to RA onset remain unclear, multiple studies offer plausible explanations. Firstly, RF-EMF may reduce the concentration and activity of antioxidants in human plasma, including glutathione, catalase, and superoxide dismutase. Concurrently, these RF-EMFs elevate lipid peroxidation levels, significantly increasing circulating pro-inflammatory cytokine concentrations: elevated IL-1β, IL-6, and TNF-α levels, thereby heightening RA risk via oxidative stress pathways [8,22]. Secondly, the 484-nanometre blue light emitted by mobile phones activates retinal ganglion cells, subsequently activating melanopsin. Melanopsin transmits light signals to the suprachiasmatic nucleus, thereby inhibiting pineal gland secretion of melatonin [10]. Melatonin stimulates the immune system by enhancing T-lymphocyte activity, generating multiple humoral immune responses. Melatonin also reduces peripheral Th1/Th17 cell ratios and inhibits expression of TNF-α, IL-1β, IL-17, and IL-6 [23,24].Therefore, blue light emitted by mobile phones may impair melatonin's ability to suppress inflammatory factors, which is positively correlated with an increased incidence of RA in the general population. However, no statistically significant association was observed between HMPU and the risk of RA onset. A plausible explanation is that using the hands-free mode reduces direct head exposure to radiofrequency electromagnetic fields (RF-EMF). Wall S et al. also found that the hands-free mode can attenuate RF-EMF intensity by two orders of magnitude. Therefore, due to the attenuation of RF-EMF, an association between HMPU and RA risk may not be observable [25]

MPU, as a form of sedentary lifestyle, offers an alternative perspective to explain its potential association with RA onset. Data on the link between prolonged sedentary behavior and RA indicate that increased self-reported sedentary time among participants is associated with a higher risk of RA development [26]. Furthermore, the results of two Mendelian randomization studies reveal a causal relationship between extended sedentary behavior and RA onset, as well as an independent genetic causal link between longer sedentary time and the risk of various types of arthritis, including RA [27,28].A plausible explanation is that MPU, as a sedentary behavior, is positively correlated with elevated levels of IL-6 in the body. IL-6 appears to activate the production of IL-17, which stimulates osteoclastogenesis through both RANKL-dependent and RANKL-independent pathways. IL-17 can also induce osteoblasts to produce RANKL, and RANKL synergizes with TNF-α to promote osteoclast formation. These factors collectively have the potential to trigger the onset of RA [29,30].

In the subgroup analysis, we found that there was an association between WMPU and RA in females, whereas no such association was observed in males. To date, there is no established biological mechanism that explains our findings. Further studies are needed to confirm our results and to further clarify the underlying biological mechanisms.

### Strengths and limitations

Our research also possesses advantages. It employs a longitudinal cohort design utilizing an extensive database, with a large amount of data, and a strong argument for causality. Furthermore, our study has several limitations. Firstly, in our study, although a series of variables have been adjusted, there may still be many unknown residual confounding factors that we have not excluded. Additionally, the study employed MPU variables measured at baseline, when mobile phones emitted higher levels of radiofrequency electromagnetic fields compared to current devices, potentially leading to an overestimation of the association between MPU and RA incidence. Finally, given that the study sample comprised 91.04% White participants with an average age of 56 years, the applicability of the findings to younger populations who typically engage in longer and more frequent mobile phone use is somewhat limited. Therefore, caution is warranted when extrapolating the results.

## Conclusions

This study found that MPU, duration of MPU, and weekly usage time were positively correlated with an increased probability of RA onset. However, the use of hands-free mode when making or receiving calls showed no significant correlation with RA incidence. Therefore, on one hand, future research could conduct randomized controlled trials (RCTs) to validate whether implementing MPU management strategies can reduce the risk of RA development. On the other hand, this study also provides a preliminary foundation and hypothesis for future investigations into the causal relationship between MPU and RA using genetic proxy tools for MPU.

## Supporting information

S1 File Assessment of mobile phone use, length of mobile phone use, weekly usage of mobile phone, and hands-free device/speakerphone use with mobile phone in the UK Biobank. (DOCX)

S2 File Assessment of covariates in the UK Biobank. (DOCX)

S1 Table Classification of four types of UKB mobile phone usage behaviours. (DOCX)

S2 Table The disease code for RA. (DOCX)

S3 Table RA medications and their codes recorded by UK Biobank participants. (DOCX)

S4 Table The number of participants with missing covariate data. (DOCX)

S5 Table Association between weekly cell phone usage and the risk of RA among different genders. (DOCX)

S6 Table Sensitivity analysis of the association between weekly cell phone usage, duration of use, and the risk of RA occurrence, excluding individuals who developed the disease in the first five years. (DOCX)

S7 Table Sensitivity analysis of the association between weekly cell phone usage, duration of use, and the risk of RA occurrence, with all missing values removed. (DOCX)

S8 Table Sensitivity analysis of the association between weekly cell phone usage, duration of use, and the risk of RA occurrence, further adjusted for pre-tax income and exercise status. (DOCX)

S9 Table Sensitivity analysis of the association between weekly cell phone usage, duration of use, and the risk of RA occurrence (RA onset defined as use of RA treatment medications). (DOCX)

S10 Table Sensitivity analysis of the association between weekly cell phone usage, duration of use, and the risk of RA occurrence (Including only RA seropositive onset cases). (DOCX)

S11 Table Sensitivity analysis of the association between weekly cell phone usage, duration of use, and the risk of RA occurrence (linear trend analysis conducted by treating the independent variable as a numerical variable). (DOCX)

S1 Fig Forest plot of the correlation between years of using mobile phones and the occurrence of RA. (TIF)

S2 Fig Forest plot of the correlation between weekly use of mobile phones and the occurrence of RA. (TIF)

S3 Fig Forest plot of the correlation between the frequency of using hands-free phones within three months and the occurrence of RA. (TIF)

S4 Fig Dose-response relationship diagram between weekly mobile phone usage duration and RA. (TIF)
