# Supplementary material for: Association between mobile phone use and risk of rheumatoid arthritis: A large prospective cohort study
Source: PLoS One. 2026 May 22;21(5):e0347330. doi: 10.1371/journal.pone.0347330 (PMC13196935; doi:10.1371/journal.pone.0347330)
Supplement: S1 File — (DOCX) [file pone.0347330.s015.docx]

**S1 Method. Assessment of Mobile Phone Use, Length of Mobile Phone Use, Weekly Usage of Mobile Phone, and Hands-Free Device/Speakerphone Use with Mobile Phone in the UK Biobank.**

Four mobile phone usage characteristics—(mobile phone use, length of mobile phone use, weekly usage of mobile phone, and hands-free device/speakerphone use with mobile phone)—were derived from responses to three touchscreen questionnaire(For approximately how many years have you been using a mobile phone at least once per week to make or receive calls?, over the last 3 months, on average how much time per week did you spend making or receiving calls on a mobile phone?, Over the last 3 months, how often have you used a hands-free device/speakerphone when making or receiving calls on your mobile?) Items completed by UKB participants at baseline (2006-2010).

Mobile phone use was defined based on the following question, ‘For approximately how many years have you been using a mobile phone at least once per week to make or receive calls?’, those answering ‘Never used mobile phone at least once per week’ were defined as mobile phone non-users, and participants who have been using a mobile phone at least once per week to make or receive calls were defined as mobile phone users. Mobile phone users were further asked for weekly usage of a mobile phone, and hands-free device/speakerphone use with mobile phones, while others did not.

Length of mobile phone use was assessed using the following question, ‘For approximately how many years have you been using a mobile phone at least once per week to make or receive calls?’, and seven options were provided to respond: ‘never used a mobile phone at least once per week’, ‘1 year or less’, ‘2-4 years’, ‘5-8 years’, ‘more than 8 years’, ‘do not know’, and ‘prefer not to answer’.

Weekly usage of mobile phone for making or receiving calls was obtained using the following question, ‘over the last 3 months, on average how much time per week did you spend making or receiving calls on a mobile phone?’, and eight options were given to respond: ‘>6 hours’, ‘4-6 hours’, ‘1-3 hours’,‘30-59 minutes’， ‘5-29 minutes’, ‘< 5 minutes’ ， ‘do not know’, and ‘prefer not to answer’.

Hands-free device/speakerphone uses with mobile phones to make or receive calls was assessed using the following question, ‘Over the last 3 months, how often have you used a hands-free device/speakerphone when making or receiving calls on your mobile?’, and seven options were given to respond: ‘never or almost never’, ‘less than half the time’, ‘about half the time’, ‘more than half the time’, ‘always or almost always’, ‘do not know’, and ‘prefer not to answer’.
The detailed variable definition method has been described elsewhere^[1]^.

The cohort analysis comprised two distinct populations: 479,966 participants with complete data for MPU and LMPU variables, and 404,383 participants with complete data for WMPU and HMPU variables. Detailed definitions are as follows:

In this study, participants who responded to the question, “For approximately how many years have you been using a mobile phone at least once per week to make or receive calls?” with any of the following five options were considered as having non-missing data for the “Length of mobile phone use” variable: “never used a mobile phone at least once per week,” “1 year or less,” “2–4 years,” “5–8 years,” or “more than 8 years.” This group totaled 479,966 participants. Those who selected “do not know” or “prefer not to answer” were treated as missing values and excluded, amounting to 15,969 exclusions (see Figure 1). Among these 479,966 participants, the 72,684 individuals who selected “never used a mobile phone at least once per week” constituted the “never” or “no” groups for both LMPU and MPU, representing non-users of mobile phones.

Among the 479,966 participants with complete data for the “Length of mobile phone use” variable, 72,684 chose “never used a mobile phone at least once per week.” These participants did not answer the subsequent questions: “Over the last 3 months, on average how much time per week did you spend making or receiving calls on a mobile phone?” and “Over the last 3 months, how often have you used a hands-free device/speakerphone when making or receiving calls on your mobile?” They were defined as non-users, leaving 407,282 participants. These remaining participants responded to both follow-up questions. For the first question, they could select from eight options: “>6 hours,” “4–6 hours,” “1–3 hours,” “30–59 minutes,” “5–29 minutes,” “<5 minutes,” “do not know,” or “prefer not to answer.” For the second question, they could choose from seven options: “never or almost never,” “less than half the time,” “about half the time,” “more than half the time,” “always or almost always,” “do not know,” or “prefer not to answer.” Participants who selected “do not know” or “prefer not to answer” for either question were considered to have missing data for either the “Weekly usage time of mobile phones for making or receiving calls” variable or the “Hands-free device/speakerphone used for making or receiving calls” variable, resulting in 2,899 exclusions. Ultimately, 404,383 participants remained with complete data for both variables.
