# Supplementary material for: Association between mobile phone use and risk of rheumatoid arthritis: A large prospective cohort study
Source: PLoS One. 2026 May 22;21(5):e0347330. doi: 10.1371/journal.pone.0347330 (PMC13196935; doi:10.1371/journal.pone.0347330)
Supplement: S2 File — (DOCX) [file pone.0347330.s016.docx]

**S2 Method. Assessment of covariates in the UK Biobank.**

We assessed several potential confounders using self-reported questionnaires and physical measurement variables. These included age (continuous variable; Field ID: 21022), calculated by subtracting the date of birth from the date of assessment; sex (female, male; Field ID: 31); education level (university degree, other; Field ID: 6138); ethnicity (white, other; Field ID: 21000); smoking status (never, current, past; Field ID: 20116); frequency of drinking alcohol (never, less than once a month, 2-4 times a month, 2-3 times a week, 4 or more times a week; Field ID: 29091); and body mass index (BMI, continuous variable, kg/m²; Field ID: 21001), and average total household income before tax (Less than 18,000, 18,000 to 30,999, 31,000 to 51,999, 52,000 to 100,000, Greater than 100,000; Field ID: 738).

Sleep quality was calculated as follows: it was first assessed using five touchscreen questionnaire items: the amount of time you sleep each day (Field ID: 1160), whether you consider yourself an early bird or a night owl (Field ID: 1180), whether you have difficulty falling asleep or waking up in the middle of the night (Field ID: 1200), whether your partner or friends complain about your snoring (Field ID: 1210), and the likelihood of you napping or falling asleep during the day when not intended (Field ID: 1220). The answers to the first question were scored as 0 points for less than 7 hours or at least 9 hours of sleep each day, and 1 point for 7 to less than 9 hours. The answers to the second question were scored as 1 point for considering oneself an early riser or preferring early hours over late hours, and 0 points for considering oneself a night owl or preferring late hours over early hours. The answers to the third question were scored as 1 point for never or sometimes, and 0 points for usually. The answers to the fourth question were scored as 1 point for no complaints and 0 points for complaints. The answers to the fifth question were scored as 1 point for never or sometimes, and 0 points for often and always. The scores from the five questions were summed to obtain a total score ranging from 0 to 5 points. A higher total score indicated better sleep quality, with low sleep quality (total score ≤ 1), medium sleep quality (2 ≤ total score ≤ 3), and excellent sleep quality (total score ≥ 4)^[2]^.

Physical activity intensity was calculated as follows: we measured the weekly metabolic equivalent (MET) per minute for each participant. The specific calculation involved collecting the time each participant spent walking (3.3 MET), doing moderate physical activities (4 MET), and strenuous physical activities (8 MET) per week. The weekly time spent on each type of physical activity was then multiplied by the respective MET value to obtain the total weekly MET (MET-min/week). Participants were categorized into three groups: low physical activity intensity (< 600 MET-minutes/week), moderate physical activity intensity (600 to < 3000 MET-minutes/week), and high physical activity intensity (≥ 3000 MET-minutes/week)^[3]^.

The quality control and genotyping process in the UK Biobank has been described elsewhere^[4]^. We also obtained the published standard polygenic risk score(PRS) for RA from the UK Biobank. The exact calculation of the PRS is described in this article^[5]^.

**References**

[1] YE Z, ZHANG Y, ZHANG Y, et al. Mobile phone calls, genetic susceptibility, and new-onset hypertension: results from 212 046 UK Biobank participants[J]. Eur Heart J Digit Health, 2023,4(3): 165-174.

[2] FAN M, SUN D, ZHOU T, et al. Sleep patterns, genetic susceptibility, and incident cardiovascular disease: a prospective study of 385 292 UK biobank participants[J]. Eur Heart J, 2020,41(11): 1182-1189.

[3] CAO Y, HU Y, LEI F, et al. Associations between leisure-time physical activity and the prevalence and incidence of osteoporosis disease: Cross-sectional and prospective findings from the UK biobank[J]. Bone, 2024,187: 117208.

[4] BYCROFT C, FREEMAN C, PETKOVA D, et al. The UK Biobank resource with deep phenotyping and genomic data[J]. Nature, 2018,562(7726): 203-209.

[5] NI J, ZHOU Q, MENG S, et al. Sleep patterns, physical activity, genetic susceptibility, and incident rheumatoid arthritis: a prospective cohort study[J]. BMC Med, 2024,22(1): 390.
