# Supplementary material for: Association between mobile phone use and risk of rheumatoid arthritis: A large prospective cohort study
Source: PLoS One. 2026 May 22;21(5):e0347330. doi: 10.1371/journal.pone.0347330 (PMC13196935; doi:10.1371/journal.pone.0347330)
Supplement: S1 Table — (DOCX) [file pone.0347330.s001.docx]

**S1 Table. Classification of four types of UKB mobile phone usage behaviours.**

| **Touchscreen questionnaire from UKB** | **Answer** | **Exposing variable names** | **Exposed variable categories** |
| --- | --- | --- | --- |
| For approximately how many years have you been using a mobile phone at least once per week to make or receive calls? |  | Mobile phone use |  |
|  | Never used mobile phone at least once per week |  | No |
|  | One year or less |  | Yes |
|  | Two to four years |  | Yes |
|  | Five to eight years |  | Yes |
|  | More than eight years |  | Yes |
| For approximately how many years have you been using a mobile phone at least once per week to make or receive calls? |  | Length of mobile phone use |  |
|  | Never used mobile phone at least once per week |  | Never |
|  | One year or less |  | ≤1 year |
|  | Two to four years |  | 2-4 years |
|  | Five to eight years |  | 5-8 years |
|  | More than eight years |  | >8 years |
| Over the last 3 months, on average how much time per week did you spend making or receiving calls on a mobile phone? |  | Weekly usage of mobile phone |  |
|  | Less than 5minutes |  | <5 min |
|  | 5-29 minutes |  | 5-29 min |
|  | 30-59 minutes |  | 30-59 min |
|  | 1-3 hours |  | 1-3 h |
|  | 4-6 hours |  | 4-6 h |
|  | More than 6 hours |  | >6 h |
| Over the last 3 months, how often have you used a hands-free device/speakerphone when making or receiving calls on your mobile? |  | Hands-free device/speakerphone use with mobile phone |  |
|  | Never or almost never |  | Never or almost never |
|  | Less than half the time |  | Less than half the time |
|  | About half the time |  | About half the time |
|  | More than half the time |  | More than half the time |
|  | Always or almost always |  | Always or almost always |
