# Supplementary material for: Association between mobile phone use and risk of rheumatoid arthritis: A large prospective cohort study
Source: PLoS One. 2026 May 22;21(5):e0347330. doi: 10.1371/journal.pone.0347330 (PMC13196935; doi:10.1371/journal.pone.0347330)
Supplement: S2 Table — (DOCX) [file pone.0347330.s002.docx]

**S2 Table. The disease code for RA.**

| **Rheumatoid arthritis** | **ICD10** | **ICD9** | **Self-report** |
| --- | --- | --- | --- |
|  | M050、M051、M052、M053、M058、M059、M060、M061、M062、M063、M064、M068、M069 | 71400、71401、71403、71404、71405、71406、71409 | 1464 |
