# Supplementary material for: Association between mobile phone use and risk of rheumatoid arthritis: A large prospective cohort study
Source: PLoS One. 2026 May 22;21(5):e0347330. doi: 10.1371/journal.pone.0347330 (PMC13196935; doi:10.1371/journal.pone.0347330)
Supplement: S5 Table — (DOCX) [file pone.0347330.s005.docx]

**S3 Table. RA medications and their codes recorded by UK Biobank participants.**

| Drug Name | UK Biobank data fields | Corresponding code |
| --- | --- | --- |
| Prescription medication  self-report past and current medicine are taken through a  interview by a trained nurse, including the following medicine:    (1) Steroids: corticosteroids, depomedrone, triamcinolone,  methylprednisolone, prednisolone, prednisone (also listed in  UK Biobank as Deltacortril enteric; Deltastab; Precortisyl;  Prednesola)    (2) Synthetic disease-modifying anti-rheumatic drugs  (DMARDs): Auranofin (also listed in UK Biobank as  Ridaura), Azathioprine (also listed in UK Biobank as Imuran),  hydroxychloroquine (also listed in UK Biobank as Plaquenil),  leflunomide (also listed in UK Biobank as Arava),  methotrexate, methotrexate injections, myocrisin,  penicillamine, sulfasalazine (also listed in UK Biobank as  Sulazine; salazopyrin; sulphasalazine)    (3) Biologic DMARDS: abatacept, adalimumab (also listed in  UK Biobank as Humira injection solution), certolizumab,  etanercept, golimumab, infliximab, rituximab, tocilizumab    (4) Nonsteroidal anti-inflammatory drugs (NSAIDs):  Indomethacin, Sulindac, Diclofenac, Etodolac, Acemetacin,  Ketorolac, Aceclofenac, Diclofenac\|Misoprostol,  Piroxicam, Tenoxicam, Meloxicam, Ibuprofen, Naproxen,  Ketoprofen, Flurbiprofen, Tiaprofenic Acid, Ibuprofen  \|Menthol, Naproxen\|Misoprostol, Mefenamic Acid,  Tolfenamic Acid, Celecoxib, Etoricoxib, Nabumetone,  Glucosamine, Chondroitin Sulfate | 20003: “Treatment/medication code” | Steroids, Synthetic DMARDs, and Biologic  DMARDS:  1140874936,1140874940,1140874944,1140874950,  1140874954,1140874956,1140874978,1140868426,  1140883058,1140883060,1140883062,1140883064,  1140874976,1140800000,1140874930,1140868364,  1140868370,1140874936,1140874940,1140875316,  1140874944,1140874950,1140875400,1140875404,  1140909864,1140869930,1141145996,1140884308,  1140875392,1141166294,1141166302,1141166304,  1141166306,1140910036,1140869848,1140853054,  1140853056,1140875304,1140875306,1140875308,  1140909702,1141188900, 1140865670,1140865668,  1141188588,1141188594        NSAIDs:  1140871354,1140871604,1140921828,11408711681  140917394,1140871180,1140923920,1140909354,  1140877872,1141182674,1140871174,11411674261,  140877874,1140871188,1140871196,1141193170,  1140875278,1140884558,1140925806,1140871266,  1140927086,1140878036,1140871672,1141169530,  1140871666,1140875346,1140926732,1140926796,  1140926794,1141187776,1140871388,1141153134,  1140871472,1140871522,1140871506,1140871238,  1140871236,1140871616,1140911748,1140871638,  1140881612,1140871542,1140871546,1140928840,  1141176668,1141176670,1141180140,1141180148,  1141180150,1141180152,1140875336,1140875338,  1141188442,1187,1140871666,1140875346,  1140926732,1140926796,1140926794 |
