# Supplementary material for: Association between mobile phone use and risk of rheumatoid arthritis: A large prospective cohort study
Source: PLoS One. 2026 May 22;21(5):e0347330. doi: 10.1371/journal.pone.0347330 (PMC13196935; doi:10.1371/journal.pone.0347330)
Supplement: S6 Table — (DOCX) [file pone.0347330.s006.docx]

**S4 Table. The number of participants with missing covariate data.**

| **Covariates** | **Total participants (N=479966)** | **Mobile phone user (N=404383)** |
| --- | --- | --- |
|  | **The number of missing data (%)** | **The number of missing data(%)** |
| Education | 85823(17.88) | 66147(16.35) |
| PRS | 15044(3.13) | 10036 (2.48) |
| BMI | 2385(0.50) | 1895 (0.47) |
| Smoking | 1532(0.32) | 1271(0.31) |
| Race | 1530 (0.32) | 1168(0.29) |
| TDI | 594(0.12) | 561(0.14) |
| Drinking | 314(0.07) | 253(0.06) |
| Sleep quality | 1(0.00) | 1(0.00) |
| Age | 0(0.00) | 0(0.00) |
| Sex | 0(0.00) | 0(0.00) |

**Note:** PRS, Polygenic Risk Score; TDI, Townsend Deprivation Index.
