# Supplementary material for: Association between mobile phone use and risk of rheumatoid arthritis: A large prospective cohort study
Source: PLoS One. 2026 May 22;21(5):e0347330. doi: 10.1371/journal.pone.0347330 (PMC13196935; doi:10.1371/journal.pone.0347330)
Supplement: S7 Table — (DOCX) [file pone.0347330.s007.docx]

**S5 Table. Association between weekly cell phone usage and the risk of RA among different genders.**

| Grouping variable | | Total/cases | *HR(95%CI)* | *P value* | Total/cases | *HR(95%CI)* | *P value* | *P for interaction* |
| --- | --- | --- | --- | --- | --- | --- | --- | --- |
| Sex** | female | |  |  | male |  |  | <0.001 |
| <30mins | 138017/2060 | | ref |  | 103999/1074 | ref |  |  |
| ≥30mins | 81574/1289 | | 1.16 (1.08-1.25) | <0.05 | 80793/654 | 1.04 (0.94-1.16) | >0.05 |  |

**: adjusted for age, BMI, Townsend deprivation index, smoking status, Frequency of alcohol intake, qualification , sleep quality,RA polygenic risk score, race plus mutually adjustments for different behaviors of using mobile phone.
