# Supplementary material for: Association between mobile phone use and risk of rheumatoid arthritis: A large prospective cohort study
Source: PLoS One. 2026 May 22;21(5):e0347330. doi: 10.1371/journal.pone.0347330 (PMC13196935; doi:10.1371/journal.pone.0347330)
Supplement: S10 Table — (DOCX) [file pone.0347330.s009.docx]

**S7 Table. Sensitivity analysis of the association between weekly cell phone usage, duration of use, and the risk of RA occurrence, with all missing values removed.**

| Mobile phone use |  |  | Crude Model | |  |  | Adjusted Model* | |
| --- | --- | --- | --- | --- | --- | --- | --- | --- |
|  | N | Cases | *HR (95% CI)* | *P Value* | N | Cases | *HR (95% CI)* | *P Value* |
| Length of mobile phone use (years) |  |  |  |  |  |  |  |  |
| never | 72684 | 951 | ref |  | 53947 | 578 | ref |  |
| ≤1 | 12971 | 208 | 1.19 (1.03-1.39) | <0.05 | 8548 | 109 | 1.22 (1.00-1.50) | <0.05 |
| 2-4 | 83863 | 1172 | 1.11 (1.02-1.21) | <0.05 | 61005 | 674 | 1.09 (0.97-1.22) | >0.05 |
| 5-8 | 147342 | 1896 | 1.14 (1.06-1.24) | <0.05 | 118534 | 1301 | 1.17 (1.06-1.30) | <0.05 |
| >8 | 163106 | 1855 | 1.16 (1.07-1.26) | <0.05 | 138617 | 1413 | 1.23 (1.11-1.36) | <0.05 |
| Weekly usage time of mobile phones for making or receiving calls |  |  |  |  |  |  |  |  |
| <5 min | 84115 | 1152 | ref |  | 65421 | 729 | ref |  |
| 5-29 min | 157901 | 1982 | 0.98 (0.91-1.06) | >0.05 | 127267 | 1366 | 1.03 (0.94-1.13) | >0.05 |
| 30-59 min | 69892 | 820 | 0.99 (0.90-1.08) | >0.05 | 57157 | 592 | 1.08 (0.97-1.20) | >0.05 |
| 1-3 h | 57969 | 703 | 1.12 (1.02-1.23) | <0.05 | 48097 | 484 | 1.15 (1.02-1.30) | <0.05 |
| 4-6 h | 16960 | 215 | 1.23 (1.06-1.43) | <0.05 | 14126 | 161 | 1.37 (1.15-1.63) | <0.05 |
| >6 h | 17546 | 205 | 1.19 (1.02-1.38) | <0.05 | 14636 | 165 | 1.43 (1.20-1.70) | <0.05 |
| Categories |  |  |  |  |  |  |  |  |
| <30 min | 242016 | 3134 | ref |  | 192688 | 2095 | ref |  |
| ≥30 min | 162367 | 1943 | 1.09 (1.02-1.15) | <0.05 | 134016 | 1402 | 1.14 (1.06-1.22) | <0.05 |

*: adjusted for age, BMI, sex, Townsend deprivation index, smoking status, Frequency of alcohol intake, qualification , sleep quality, race, RA polygenic risk score.
