# Supplementary material for: Association between mobile phone use and risk of rheumatoid arthritis: A large prospective cohort study
Source: PLoS One. 2026 May 22;21(5):e0347330. doi: 10.1371/journal.pone.0347330 (PMC13196935; doi:10.1371/journal.pone.0347330)
Supplement: S11 Table — (DOCX) [file pone.0347330.s010.docx]

**S11 Table. Sensitivity analysis of the association between weekly cell phone usage, duration of use, and the risk of RA occurrence (linear trend analysis conducted by treating the independent variable as a numerical variable).**

| Mobile phone use |  |  | Crude Model | | Mobile phone use | | Adjusted Model* | |
| --- | --- | --- | --- | --- | --- | --- | --- | --- |
|  | N | Cases | *HR (95% CI)* | *P Value* |  |  | *HR (95% CI)* | *P Value* |
| Length of mobile phone use (years) |  |  |  |  | Length of mobile phone use (continuous variable,$\bar{x}\pm s$) | |  |  |
| never | 72684 | 951 | ref |  |  |  |  |  |
| ≤1 | 12971 | 208 | 1.19 (1.03-1.39) | <0.05 |  |  |  |  |
| 2-4 | 83863 | 1172 | 1.11 (1.02-1.21) | <0.05 |  |  |  |  |
| 5-8 | 147342 | 1896 | 1.14 (1.06-1.24) | <0.05 |  |  |  |  |
| >8 | 163106 | 1855 | 1.16 (1.07-1.26) | <0.05 |  |  | 1.05 (1.03-1.08) | <0.05 |
| Total | 479966 | 6082 |  |  | 2.66±1.37 | |  |  |
| Weekly usage time of mobile phones for making or receiving calls |  |  |  |  | Weekly usage time of mobile phones for making or receiving calls | |  |  |
| <5 min | 84115 | 1152 | ref |  |  |  |  |  |
| 5-29 min | 157901 | 1982 | 0.98 (0.91-1.06) | >0.05 |  |  |  |  |
| 30-59 min | 69892 | 820 | 0.99 (0.90-1.08) | >0.05 |  |  |  |  |
| 1-3 h | 57969 | 703 | 1.12 (1.02-1.23) | <0.05 |  |  |  |  |
| 4-6 h | 16960 | 215 | 1.23 (1.06-1.43) | <0.05 |  |  |  |  |
| >6 h | 17546 | 205 | 1.19 (1.02-1.38) | <0.05 |  |  |  |  |
| Categories |  |  |  |  |  |  |  |  |
| <30 min | 242016 | 3134 | ref |  |  |  |  |  |
| ≥30 min | 162367 | 1943 | 1.09 (1.02-1.15) | <0.05 |  |  | 1.07 (1.04-1.10) | <0.05 |
| Total | 404383 | 5077 |  |  | 1.55±1.31 | |  |  |

*: adjusted for age, BMI, sex, Townsend deprivation index, smoking status, Frequency of alcohol intake, qualification , sleep quality, race ,RA polygenic risk score.
